# Supplementary figures and images for: PGRMC1 effects on metabolism, genomic mutation and CpG methylation imply crucial roles in animal biology and disease
Source: BMC Mol Cell Biol. 2020 Apr 15;21:26. doi: 10.1186/s12860-020-00268-z (PMC7160964; doi:10.1186/s12860-020-00268-z)

Supplementary Figures

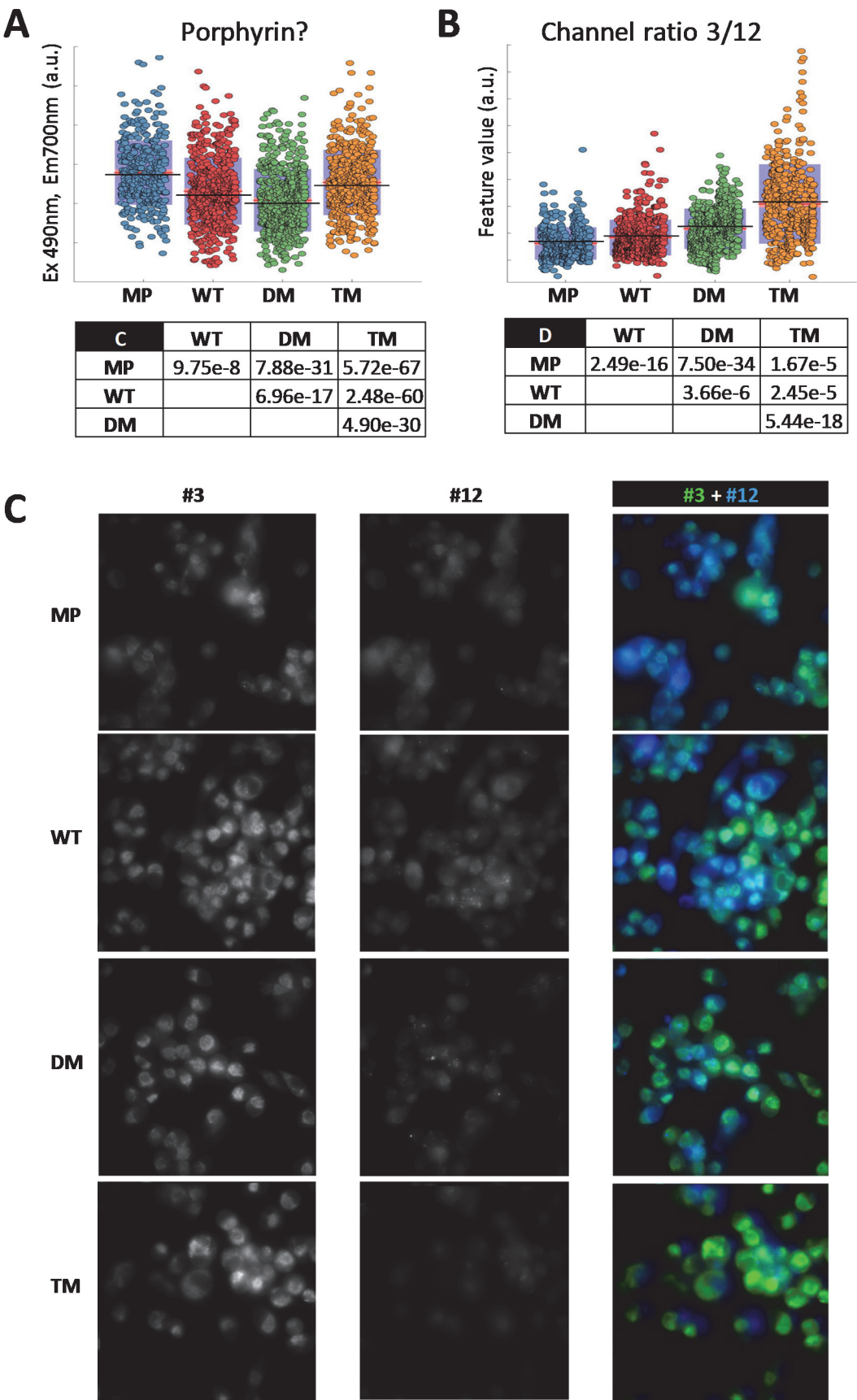

Fig. S1.

Supplement: Supplementary file 1 — Additional file 1 Fig. S1. Representative hyperspectral autofluorescence cell images from the measurements. Related to Fig. 1. (A) Mean cellular intensity of hyperspectral autofluorescence channel 18 [495 nm(Ex), 700 nm(Em)], which may reflect porphyrin or protein-bound red-shifted flavin emission [46], is significantly affected by PGRMC1-HA phosphorylation status. The table provides Kolmogorov-Smirnov test p values from pair wise comparisons. (B) The ratio of hyperspectral autofluorescence channels 3 [375 nm(Ex), 450 nm(Em)] to channel 12 [435 nm(Ex), 587 nm(Em)] differs significantly between cells. The table follows C. (C) Individual channels #3 and #4 from (B) as listed in Table S1 (left) and the same two channels superimposed (right). [file 12860_2020_268_MOESM1_ESM.pdf]

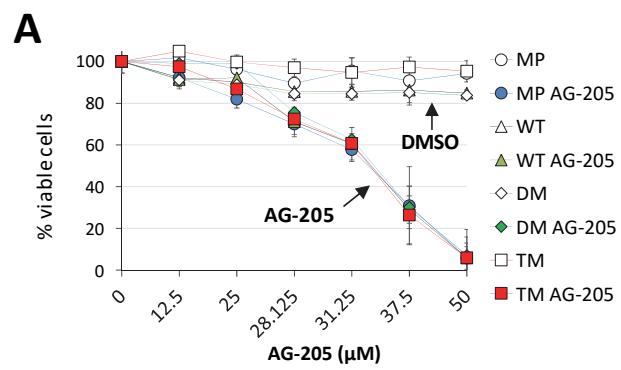

**Fig. S2.**

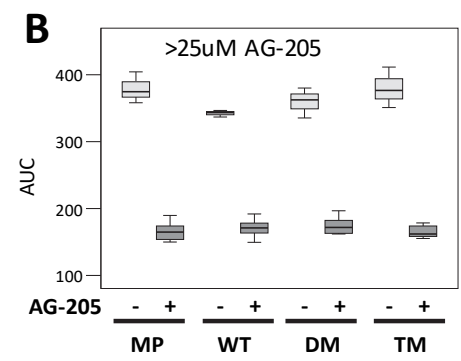

Supplement: Supplementary file 2 — Additional file 2 Fig. S2. PGRMC1 phosphorylation mutants do not affect AG-205-induced death. Related to Fig. 2. (A) AG-205-induced cell death is unaffected by PGRMC1 phosphorylation status. Cells were incubated in the presence of the indicated AG-205 concentrations (n = 8: 3x line 1, 3x line 2, 2 x line 3) or DMSO vehicle control (n = 3: 1x each cell line), and percentage viable cells was calculated relative to untreated cell controls (n = 9: 3 replicates per cell line) using MTT assay. (B) AUC results for values from A greater than 25 μM reveal no significant differences in response to AG-205 treatment between cell lines (p > 0.85, post-hoc Bonferroni after 1 way ANOVA for AG-205 treatment). The apparently greater survival of DM cells at 25 μM AG-205 observed in this panel was never observed in multiple other repeat experiments. [file 12860_2020_268_MOESM2_ESM.pdf]

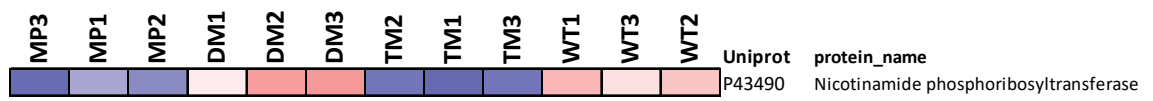

**Fig. S3.**

Supplement: Supplementary file 3 — Additional file 3 Fig. S3. SWATH-MS proteomic quantification of Nicotinamide phosphoribosyltransferase. Related to Fig. 6. The figure shows the abundance profile of P43490 nicotinamide phosphoribosyltransferase (NAMPT) from the SWATH-MS proteomics quantification of the accompanying manuscript [10]. [file 12860_2020_268_MOESM3_ESM.pdf]

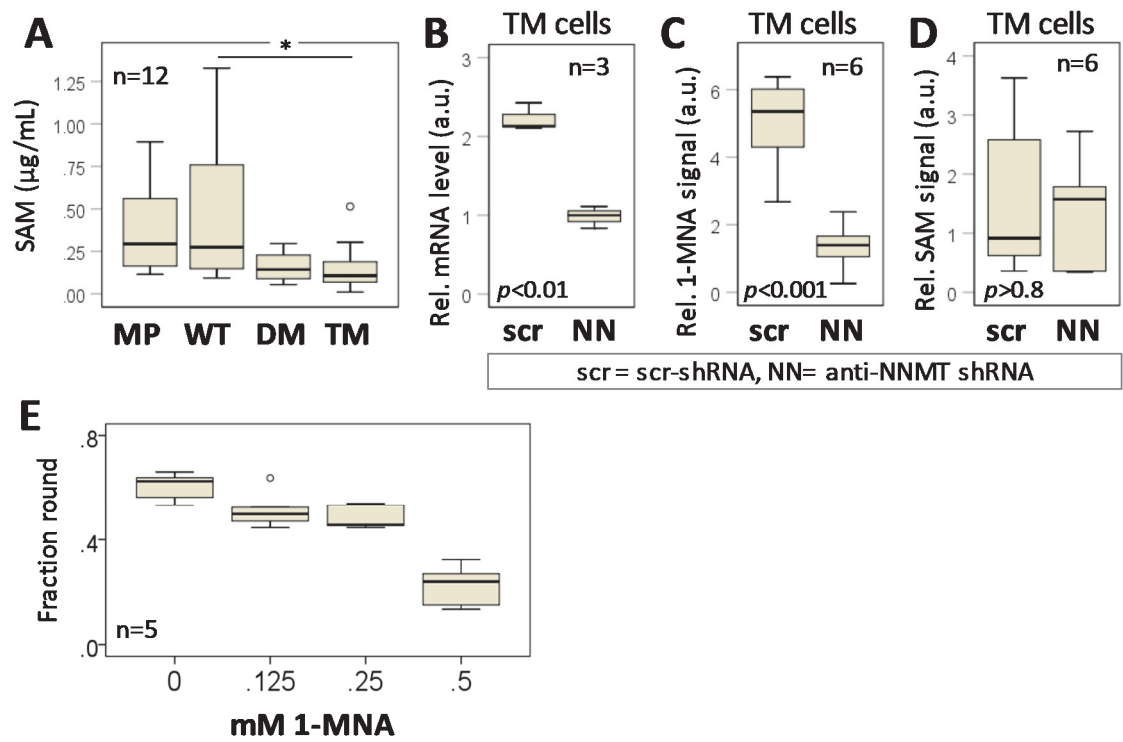

**Fig. S4.**

Supplement: Supplementary file 4 — Additional file 4 Fig. S4. PGRMC1 phosphorylation status affects the NNMT pathway. Related to Fig. 6. (A) Metabolomics quantification of S-adenosyl-Methionine (SAM) levels for the indicated cell lines, representing n = 12 (four technical replicates for each of three independent cell lines per PGRMC1 condition). (B) RT-PCR quantification of NNMT mRNA levels after treatment by an NNMT-specific shRNA (NN) or a random scramble shRNA control (scr) in TM cells stably expressing lentiplasmid-driven shRNAs. RT-PCR Methods follow A. p < 0.003 (2-tailed T test). Methods follow Fig. 6b. (C) 1-MNA ion intensities in cells from B, determined following methods from Fig. 6a, using two technical replicates of each of three biological replicates per cell condition (n = 6). Labels follow B. The result was significantly different by T-test (p < 0.001) after removal of one Scr outlier (panel displayed) or by Mann-Whitney U test (p < 0.002) including the outlier. (D) SAM ion intensities in the cells from B were not significantly different (T-test). Labels follow B. (E) 1-MNA attenuates rounded morphology in DM cells. Results for n = 5 for each 1-MNA concentration are shown in the boxplot. 0.5 mM 1-MNA was significantly different (p < 0.0001, ANOVA, post hoc Tukey HSD) to all other treatments. [file 12860_2020_268_MOESM4_ESM.pdf]

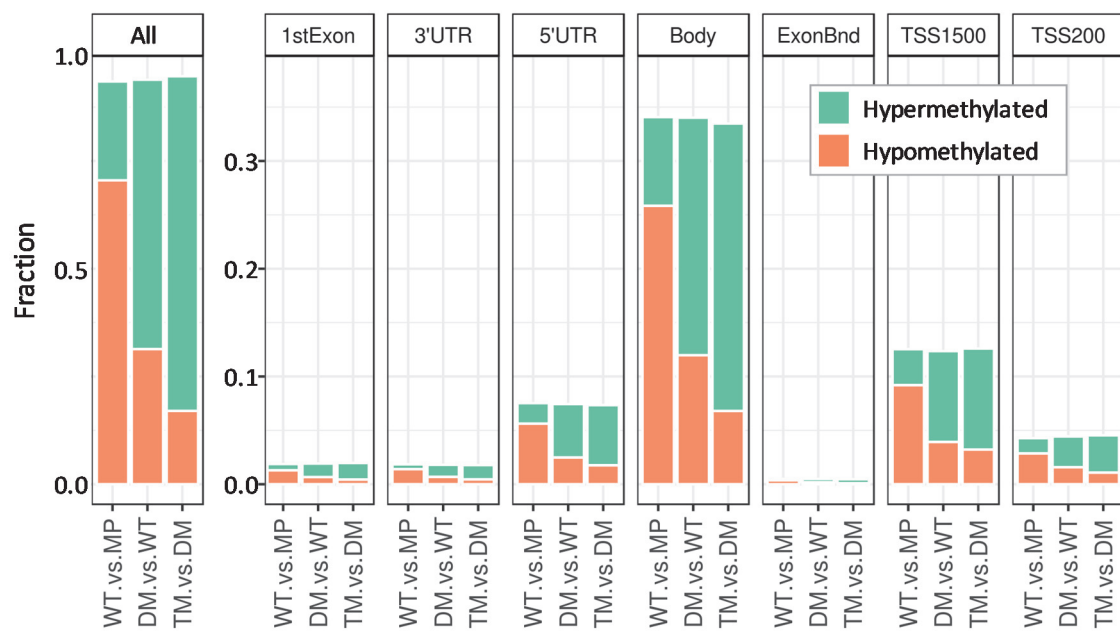

**Fig. S5.**

Supplement: Supplementary file 5 — Additional file 5 Fig. S5. Methylation status of CpG associated with annotated coding genes. Related to Fig. 7. Significant differentially methylated probes were annotated with the UCSC gene feature and the proportion of probes attributed to each feature counted. The y-axis shows the frequency of hypermethylated-hypomethylated probes per feature, and the x-axis shows the difference in those frequencies between WT/MP, DM/WT and TM/DM. 3’UTR: 3′ untranslated region; 5’UTR: 5′ untranslated region; Body: Between the ATG and stop codon; irrespective of the presence of introns, exons, TSS, or promoters; ExonBnd: exon boundaries: TSS1500/TSS200: within indicated number of residues of transcription start site (TSS). [file 12860_2020_268_MOESM5_ESM.pdf]

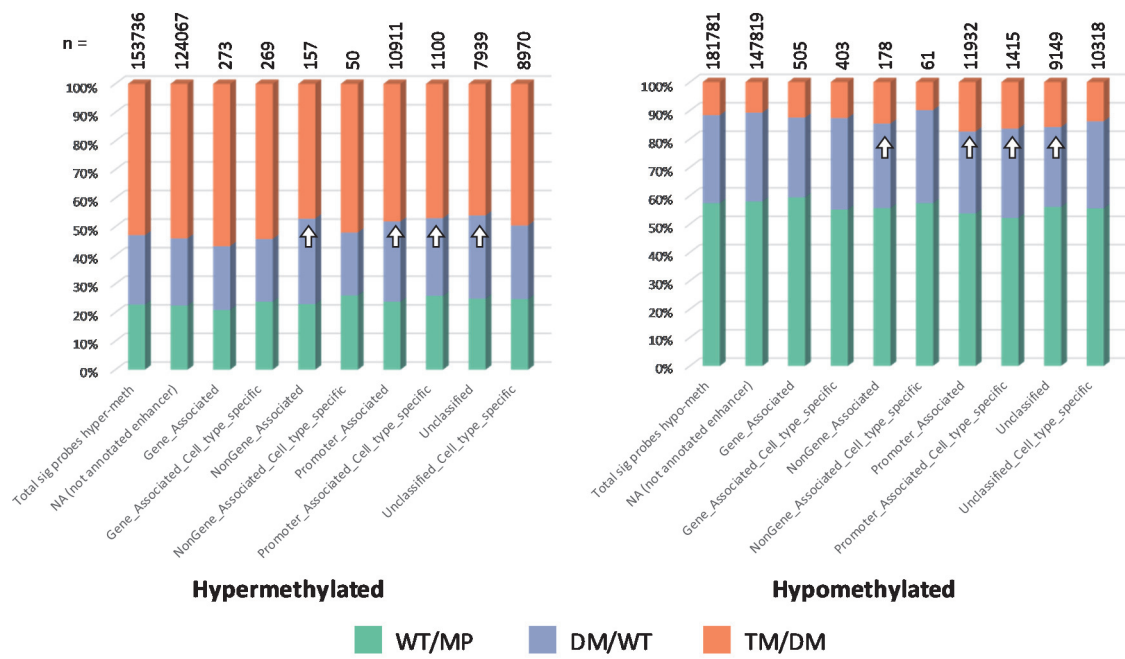

**Fig. S6.**

Supplement: Supplementary file 6 — Additional file 6 Fig. S6. Methylation status of CpG associated with annotated enhancers. Related to Fig. 7. Significant differentially methylated probes were annotated with the 450k_Enhancer feature and the proportion of probes attributed to each feature counted. Arrows indicate increased hypomethylation and reduced hypermethylation of enhancers in the TM/DM comparison. [file 12860_2020_268_MOESM6_ESM.pdf]

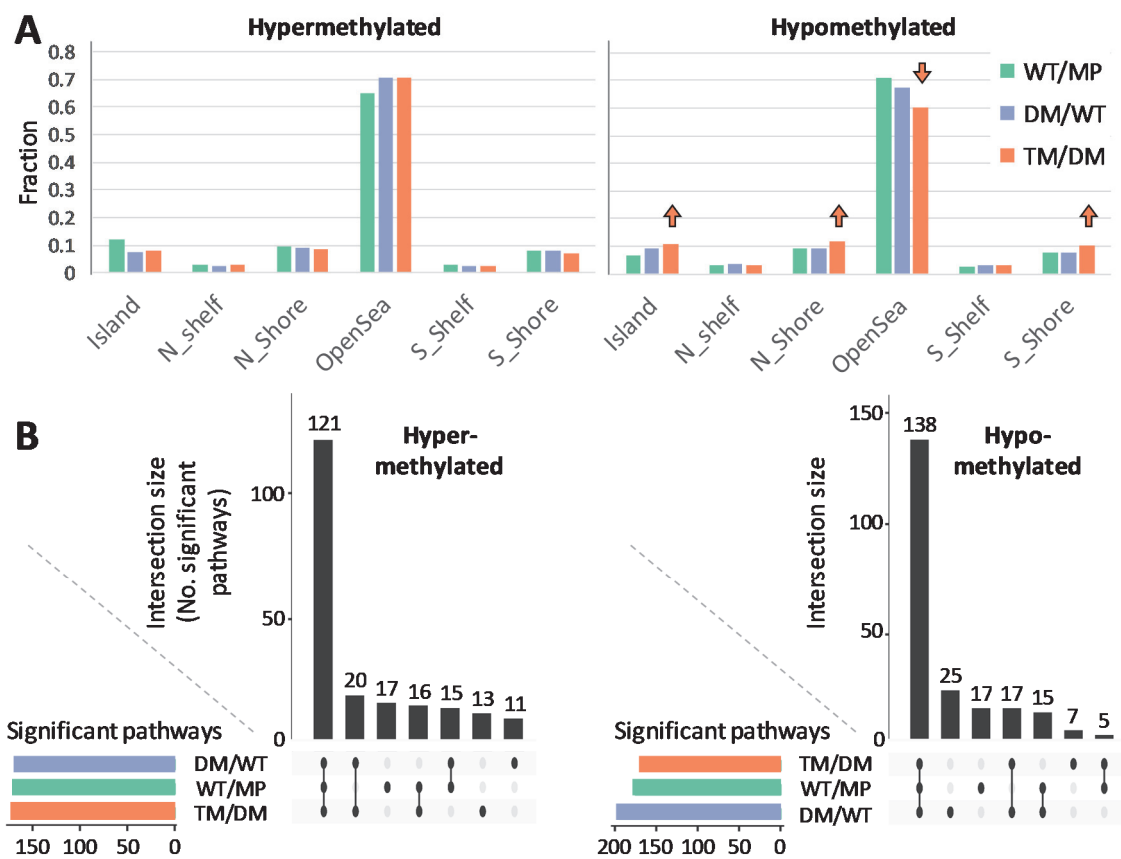

**Fig. S7.**

Supplement: Supplementary file 7 — Additional file 7 Fig. S7. Chromosomal context of differentially methylated CpG, and biological association of genes. Related to Fig. 7. (A) CpG feature classifications for differentially methylated probes. The percentage of all significant probes that were hypermethylated or hypomethylated per CpG feature is presented for each comparison. Up arrows point to relatively greater fractions of Island and Shore genomic regions for the TM/DM comparison. The down arrow points to reduced OpenSea hypomethylation in the TM/DM comparison. (B) Intersections of significant KEGG pathways between WT/MP, DM/WT and TM/DM. KEGG enrichment was applied separately to significant hypermethylated and hypomethylated probes. The main bar chart presents the number of pathways common to each comparison. Each bar represents an intersect notated by linked dots on the x-axis. The number of significant pathways per comparison is presented as a horizontal bar chart. The KEGG results are in File S1 and the list of pathways unique to each comparison are provided. Pathways analysis of probes genes corresponding to the top 2000 most variable differentially methylated probes in CpG Island or Shore chromosomal regions. Probes were separated into hypermethylated and hypomethylated data sets from D, including only probes from Island or Shores. Enriched Gene Ontology (GO) pathways enrichments are shown. The identities of pathways uniquely differential to one of the three cell type comparisons (WT/MP, DM/WT, or TM/DM) for both hypo- and hyper-methylated data sets and KEGG pathways are available in Supplemental Information File 1. [file 12860_2020_268_MOESM7_ESM.pdf]
