# Supplementary material for: PGRMC1 effects on metabolism, genomic mutation and CpG methylation imply crucial roles in animal biology and disease
Source: BMC Mol Cell Biol. 2020 Apr 15;21:26. doi: 10.1186/s12860-020-00268-z (PMC7160964; doi:10.1186/s12860-020-00268-z)
Supplement: Supplementary file 8 — Additional file 8 Table S1. Spectral channel filters employed for hyperspectral autofluorescence imaging. Related to Fig. 1. For further details of this approach see Gosnell et al. [44]. [file 12860_2020_268_MOESM8_ESM.docx]

| Channel | Excitation (Ex)/nm +/- 5 nm | Emission (Em)/nm |
| --- | --- | --- |
| 1 | 335 | 450 +/- 30 nm |
| 2 | 365 | 450+/- 30 nm |
| 3 | 375 | 450+/- 30 nm |
| 4 | 335 | 587 +/- 17.5 nm |
| 5 | 365 | 587+/- 17.5 nm |
| 6 | 375 | 587+/- 17.5 nm |
| 7 | 385 | 587+/- 17.5 nm |
| 8 | 395 | 587+/- 17.5 nm |
| 9 | 405 | 587+/- 17.5 nm |
| 10 | 415 | 587+/- 17.5 nm |
| 11 | 425 | 587+/- 17.5 nm |
| 12 | 435 | 587+/- 17.5 nm |
| 13 | 455 | 587+/- 17.5 nm |
| 14 | 470 | 587+/- 17.5 nm |
| 15 | 495 | 587+/- 17.5 nm |
| 16 | 405 | 700 long pass |
| 17 | 455 | 700 long pass |
| 18 | 495 | 700 long pass |

Table S1. Spectral channel filters employed for hyperspectral autofluorescence imaging. Related to Fig. 1. For further details of this approach see Gosnell et al. [44].
