# Supplementary material for: PGRMC1 effects on metabolism, genomic mutation and CpG methylation imply crucial roles in animal biology and disease
Source: BMC Mol Cell Biol. 2020 Apr 15;21:26. doi: 10.1186/s12860-020-00268-z (PMC7160964; doi:10.1186/s12860-020-00268-z)
Supplement: Supplementary file 9 — Additional file 9 Table S2. GO pathways enrichment results. Related to Fig. 7. The top ten GO enrichments for each cell comparison from Fig. 7. Full results are available in File S1. [file 12860_2020_268_MOESM9_ESM.docx]

| **Comparison** | **GO_ID** | **FDR** | **PValue** | **sigGO.Term** |
| --- | --- | --- | --- | --- |
| MP/WT | GO:0032501 | 8.1E-07 | 1.85E-10 | multicellular organismal process |
|  | GO:0048731 | 2.03E-06 | 9.28E-10 | system development |
|  | GO:0007275 | 5.75E-05 | 4.09E-08 | multicellular organism development |
|  | GO:0030154 | 5.75E-05 | 6.05E-08 | cell differentiation |
|  | GO:0048869 | 5.75E-05 | 6.58E-08 | cellular developmental process |
|  | GO:0048856 | 0.00013 | 1.79E-07 | anatomical structure development |
|  | GO:0022610 | 0.000141 | 2.26E-07 | biological adhesion |
|  | GO:0032502 | 0.00017 | 3.52E-07 | developmental process |
|  | GO:0023052 | 0.00017 | 3.67E-07 | signaling |
|  | GO:0009653 | 0.00017 | 3.89E-07 | anatomical structure morphogenesis |
| WT/DM | GO:0032501 | 1.79E-07 | 4.14E-11 | multicellular organismal process |
|  | GO:0009653 | 2.27E-05 | 1.05E-08 | anatomical structure morphogenesis |
|  | GO:0048731 | 5.71E-05 | 4.5E-08 | system development |
|  | GO:0050896 | 5.71E-05 | 5.28E-08 | response to stimulus |
|  | GO:0023052 | 6.44E-05 | 7.44E-08 | signaling |
|  | GO:0007165 | 0.000153 | 2.12E-07 | signal transduction |
|  | GO:0007154 | 0.000227 | 3.7E-07 | cell communication |
|  | GO:0051239 | 0.000227 | 4.2E-07 | regulation of multicellular organismal process |
|  | GO:0051716 | 0.000241 | 5.01E-07 | cellular response to stimulus |
|  | GO:0007275 | 0.000325 | 7.51E-07 | multicellular organism development |
| TM/DM | GO:0032501 | 2.71E-07 | 6.56E-11 | multicellular organismal process |
|  | GO:0048731 | 4.52E-05 | 2.19E-08 | system development |
|  | GO:0009653 | 5.33E-05 | 3.91E-08 | anatomical structure morphogenesis |
|  | GO:0007275 | 5.33E-05 | 5.16E-08 | multicellular organism development |
|  | GO:0007399 | 0.000113 | 1.37E-07 | nervous system development |
|  | GO:0050896 | 0.000141 | 2.16E-07 | response to stimulus |
|  | GO:0032502 | 0.000141 | 2.39E-07 | developmental process |
|  | GO:0048856 | 0.000173 | 3.36E-07 | anatomical structure development |
|  | GO:0030154 | 0.000214 | 4.67E-07 | cell differentiation |
|  | GO:0048869 | 0.000365 | 8.84E-07 | cellular developmental process |

Table S2. GO pathways enrichment results. Related to Fig. 7. The top ten GO enrichments for each cell comparison from Fig. 7. Full results are available in File S1.
