# Supplementary material for: PGRMC1 effects on metabolism, genomic mutation and CpG methylation imply crucial roles in animal biology and disease
Source: BMC Mol Cell Biol. 2020 Apr 15;21:26. doi: 10.1186/s12860-020-00268-z (PMC7160964; doi:10.1186/s12860-020-00268-z)
Supplement: Supplementary file 10 — Additional file 10 Table S3. Unique KEGG pathways detected for TM/DM hyper and hypo-methylated gene sets. Related to Fig. 7. The identities of the 11 hypermethylated and 7 hypomethylated pathways unique to the TM/DM comparison from Fig. S7B are given. Full results are available in File S1. [file 12860_2020_268_MOESM10_ESM.docx]

| **Data set** | **PathwayID** | **Pathway** |
| --- | --- | --- |
| Hypermeth | path:hsa04145 | Phagosome |
|  | path:hsa03050 | Proteasome |
|  | path:hsa05150 | Staphylococcus aureus infection |
|  | path:hsa05133 | Pertussis |
|  | path:hsa00983 | Drug metabolism - other enzymes |
|  | path:hsa05130 | Pathogenic Escherichia coli infection |
|  | path:hsa03410 | Base excision repair |
|  | path:hsa03430 | Mismatch repair |
|  | path:hsa04964 | Proximal tubule bicarbonate reclamation |
|  | path:hsa05014 | Amyotrophic lateral sclerosis (ALS) |
|  | path:hsa04979 | Cholesterol metabolism |
|  | path:hsa04216 | Ferroptosis |
|  | path:hsa00051 | Fructose and mannose metabolism |
| Hypometh | path:hsa05203 | Viral carcinogenesis |
|  | path:hsa04672 | Intestinal immune network for IgA production |
|  | path:hsa04657 | IL-17 signaling pathway |
|  | path:hsa00561 | Glycerolipid metabolism |
|  | path:hsa04977 | Vitamin digestion and absorption |
|  | path:hsa01230 | Biosynthesis of amino acids |
|  | path:hsa00920 | Sulfur metabolism |

Table S3. Unique KEGG pathways detected for TM/DM hyper and hypo-methylated gene sets. Related to Fig. 7. The identities of the 11 hypermethylated and 7 hypomethylated pathways unique to the TM/DM comparison from Fig. S7B are given. Full results are available in File S1.
